# The Mater CYMHS Inpatient Unit program for the management of eating disorders

**DOI:** 10.1186/2050-2974-1-S1-O14

**Published:** 2013-11-14

**Authors:** Emma Jury, Michelle George, Suzi Scholey

**Affiliations:** 1 Mater Health Services, Australia; 2 Previously of Mater Health Services, Australia

## 

In October 2011 the Mater Child and Youth Mental Health Inpatient Unit developed and implemented the Mater CYMHS Program for the Management of Eating Disorders (the protocols). The protocols were developed to improve admission and discharge pathways for young people with restrictive eating disorders as well as to improve consistency and efficacy of inpatient treatment which supports various outpatient models of care. Currently there exists little evidence for best practice treatment of restrictive eating disorders in children and adolescents on a psychiatric inpatient unit. This presentation aims to fill in the gaps in the existing literature by presenting outcome data for young people treated under the protocols. These outcomes will then be compared with those patients who received "treatment as usual" prior to the implementation of the protocols. Furthermore, we will present data examining staff perceptions of patient care of those with restrictive eating disorders both before and after the protocols were introduced.

This abstract was presented in the **Care in Inpatient and Community Settings** stream of the 2013 ANZAED Conference.

